# Avenanthramide-C reduces the viability of MDA-MB-231 breast cancer cells through an apoptotic mechanism

**DOI:** 10.1186/s12935-017-0464-0

**Published:** 2017-10-18

**Authors:** Jordan Hastings, Jason Kenealey

**Affiliations:** Department of Nutrition, Dietetics, and Food Science, Brigham Young University, ESC S249, Provo, UT 84602 USA

**Keywords:** Avenanthramides, Oats, Breast cancer, Apoptosis

## Abstract

**Background:**

Avenanthramides (AVN) are a relatively unstudied family of phytochemicals that could be novel chemotherapeutics. These compounds, found in oats, are non-toxic to healthy cells and have been shown to reduce viability of human colon and liver cancers in vitro. However, these studies do not elucidate a molecular mechanism for individual AVN. In this study we aim to see the effects of AVN on MDA-MB-231 breast cancer cells.

**Methods:**

An MTT assay was used to determine cell viability. Staining and analysis with a flow cytometer was used to identify cell cycle progression and apoptosis. FloJo software was used to analyze the cytometric data. In all experiments, statistical significance was determined by a two-tailed t test.

**Results:**

This study demonstrates that AVN-A, B, and C individually reduce viability in the MDA-MB-231 breast cancer cell line. AVN-C has the most potent decrease in tumor cell viability, decreasing viable cells to below 25% at 400 µM when compared to control after 96 h. We demonstrate that treatment with AVN-C causes DNA fragmentation and accumulation of over 90% of cells into a sub G_1_ cell cycle population. Further, we conclude that AVN-C treated cells activate apoptosis because 97% of treated cells stain positive for annexin V while 91% have caspase-3/7 activity, a late marker of apoptosis.

**Conclusions:**

Breast cancer cells treated with AVN-C have a decrease in cell viability, an increase in the sub G_1_ population, and stain positive for both annexin V and caspase activity, indicating that AVN-C induces apoptosis in breast cancer cells. These compounds may be able to act as chemotherapeutics as demonstrated through future in vivo studies.

## Background

In the US, nearly one in eight women will develop invasive breast cancer over the course of their life. In 2017, breast cancer is expected to account for nearly 30% of newly diagnosed cancers in woman, and result in over 40,000 deaths. For US women, breast cancer has a higher death rate than any other cancer, besides lung cancer [1]. Over the past two decades, scientists have determined the mechanistic characteristics of cancer as outlined in the ‘Hallmarks of Cancer’ described by Hanahan and Weinberg in 2000 and later added to in 2011; some of these hallmarks include self-sufficiency in growth signaling, insensitivity to anti-growth signals, evading apoptosis, genome instability and mutation, and tumor promoting inflammation [2, 3].

**Fig. 1:**
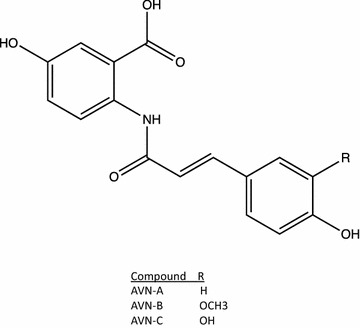
The structure of AVN-A, B and C

Avenanthramides (AVN) are a family of phytochemicals found in oats. There are three main isoforms of the chemical simply classified as avenanthramide A, B, and C (Fig. 1) [4]. Currently available literature demonstrates that AVN have chemotherapeutic potential due to its effects on some of the hallmarks of cancer. First, AVN have anti-inflammatory properties [5] and by modulating pro-inflammatory cytokines AVN could modulate the tumor microenvironment and decrease tumor size. Additionally, AVN can affect cell cycle progression and have been shown to arrest cells by decreasing cyclins [6, 7]. The ability to affect cyclin levels, along with documented decreases in viability have led to the understanding that AVN-C is anti-proliferative [8, 9]. Alternatively, a mixture of all three AVN isoforms activated pro-apoptotic mechanisms such as caspases, but the ability of each AVN isoform to induce apoptosis has not been evaluated [10].

Current cancer treatments, though moderately effective, attack both cancerous and non-cancerous cells, resulting in dose-limiting side effects. Conversely, diets consisting of AVN enriched oat extract have shown no toxicity in both animal and human models [11]. In addition, unlike many other plant-based molecules, AVN have high bioavailability, making them potential chemotherapeutics. In human trials the maximum plasma concentration (C_max_) of AVN-A, B, and C have been reported at a range of 166.7–1002.2 nmol/L for AVN-A, 49.3–153.5 nmol/L for AVN-B, and 29.6–328.1 nmol/L for AVN-C when participants consumed 1 g of avenanthramide enriched oat extract which contained 154, 109, and 117 μM/g of AVN-A, B and C respectively [12]. Notably, when ingesting half the amount of AVN C_max_ fell more than half, suggesting that higher levels of consumption may lead to even higher C_max_ levels.

In this study, we tested the effects of AVN A, B, and C on MDA-MB-231 breast cancer cells and found that all three AVN decrease breast cancer cell viability in a time and concentration dependent manner; AVN-C is the most potent of the three compounds. Furthermore, we demonstrate that the decrease in viability induced by AVN-C occurs through an apoptotic mechanism as measured through multiple methods of flow cytometric analysis.

## Materials and methods

Avenanthramides A, B, and C were purchased from Sigma Aldrich (St. Louis, MO) and stocks were diluted to 60 mM by DMSO. MDA-MB-231 human breast cancer (HTB-26) cell line was purchased from ATCC (Manassas, VA). The passage numbers of cells used in experiments ranged from 5 to 25. MTT (3-(4,5-dimethylthiazol-2-yl)-2,5-diphenyltetrazolium bromide) was purchased from Acros Organics (Morris Plains, NJ). MTT solvent was made up in the lab using 4 mM HCl, 0.1% Nonidet P-4 (NP40) in isoproponol. Propidium iodide (PI) was purchased from Cayman Chemical (Ann Arbor, MI). FITC Annexin V Apoptosis Detection Kit (556547) was purchased from BD Biosciences (San Jose, CA). CellEvent Caspase 3/7 Green Flow Cytometry Assay Kit was purchased from Thermo Fisher Scientific (Waltham, MA) (C10427).

### Cell culture

MDA-MB-231 cells were cultured in Dulbecco’s Modified Eagle Medium (DMEM). DMEM was supplemented with 10% heat-inactivated fetal bovine serum (FBS) and 1% penicillin/streptomycin. The cell line was cultured at 37 °C in 5% CO_2_. Cells were subcultured by treatment with trypsin upon reaching near confluency and reseeded by placing one million cells into a fresh T-75 culture plate.

### Cell viability analysis

Cell viability was determined using an MTT assay. Cell were plated at 10,000 cells per well in a 96-well plate. Cells were incubated for 24 h at 37 °C to allow for adhesion, at which time the DMEM was removed and new media with individual avenanthramide analogues at the indicated concentration was added. After the indicated treatment period was reached (48–96 h) 20 µL of 5 mg/mL MTT was added to each well. Cells were again incubated for 3.5 h at 37 °C in a 5% CO_2_ culture hood. The media and MTT was removed and 150 µL of MTT solvent was added. Cells were agitated on an orbital shaker at 75 rpm for 15 min before absorbance was read at 590 nm with a reference filter at 620 nm.

### Cell cycle analysis

Cells were plated at 100,000 cells per well in a 12-well plate and allowed to adhere for 24 h. DMEM was removed and new culture media with the indicated concentrations of each avenanthramide was added. After a 96-h treatment period cells were collected and pelleted down. Pellets were washed once with 1× PBS. 500 µL of 70% ethanol was added dropwise while vortexing and cells were placed in 4 °C overnight. The cells were then pelleted down and the ethanol was removed. The pellet was washed with 1× PBS before adding 200 µL PI made up at 50 μg/mL in 1× PBS. Samples were analyzed with a BD Accuri Flow Cytometer for 50,000 counts.

### Annexin V and PI apoptosis analysis

Cells were plated at 100,000 cells per well in a 12-well plate and allowed to adhere for 24 h. DMEM was removed and new culture media with the indicated concentrations of each avenanthramide was added. After a 96-h treatment period cells were harvested and subjected to centrifugation. Staining was performed per the manufacturer’s instructions (BD Biosciences). Briefly, cells were pelleted down and washed twice with cold PBS before being resuspended in 1× binding buffer at 1 × 10^6^ cells/mL. 5 μL FITC annexin V and PI were added to each sample. Samples were gently vortexed then incubated for 15 min in the dark before 400 µL 1× binding buffer was added to each tube. Samples were analyzed with a BD Accuri Flow Cytometer for 50,000 counts.

### CellEvent caspase 3/7 green flow cytometry apoptosis analysis

Cells were plated at 50,000 cells per well in a 12-well plate and allowed to adhere for 24 h. DMEM was removed and new culture media with indicated concentrations of each avenanthramide was added. After 96 h treatment period media and cells were harvested and centrifuged down. Fluorescent labels were added per the manufacturer’s instructions (Thermo Fisher). Briefly, 1 µL of CellEvent Caspase-3/7 Green Detection Reagent was added to all samples which were then incubated for 30 min. During the last 5 min of incubation 1 µL of the SYTOX AADvanced dead cell stain solution was added to each sample. Samples were analyzed with a BD Accuri Flow Cytometer for 50,000 counts.

### Analysis of flow cytometric data

Data obtained from the flow cytometer was analyzed with FlowJo software using unstained cells and cell samples stained with one marker only to set fluorescence compensations for each assay.

### Statistical significance

In all experiments, statistical significance was determined by a two-tailed t test. Significant differences in cell viability between DMSO negative control vehicle and treatment (either AVN or staurosporine positive control for activating apoptosis) are indicated by * if p < 0.05, ** if p < 0.01 and *** if p < 0.001. For cell viability experiments significance was determined between vehicle and each AVN concentration after normalization. For cell cycle analysis experiments significance was determined by comparing each stage of the cell cycle after AVN treatment with the same stage from each vehicle control treatment. For all quadrant based flow cytometry experiments significance was determined by normalizing the quadrants and then comparing each vehicle quadrant to its treated counterpart.

## Results

### Avenanthramides decrease breast cancer viability in vitro

**Fig. 2:**
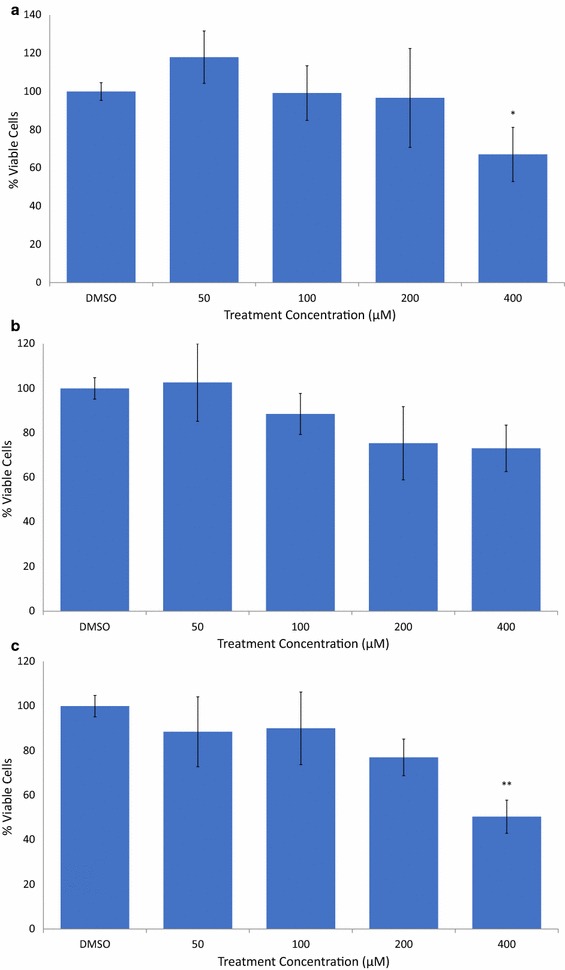
AVN-A decrease viability of MDA-MB-231 breast cancer cells in a time and concentration dependent manner. **a** MTT cell viability assay after treatment with DMSO 0.4% (vehicle) or AVN-A for 48 h at the indicated concentrations. **b** AVN-A treatment, 72 h. **c** AVN-A treatment, 96 h. Averages of a biological replicate done in triplicate; * indicates statistical significance difference from 0.4% DMSO negative control as determined by a t test at p < 0.05 level of significance, ** indicates significance level at p < 0.01, *** indicates significance at p < 0.001 level

**Fig. 3:**
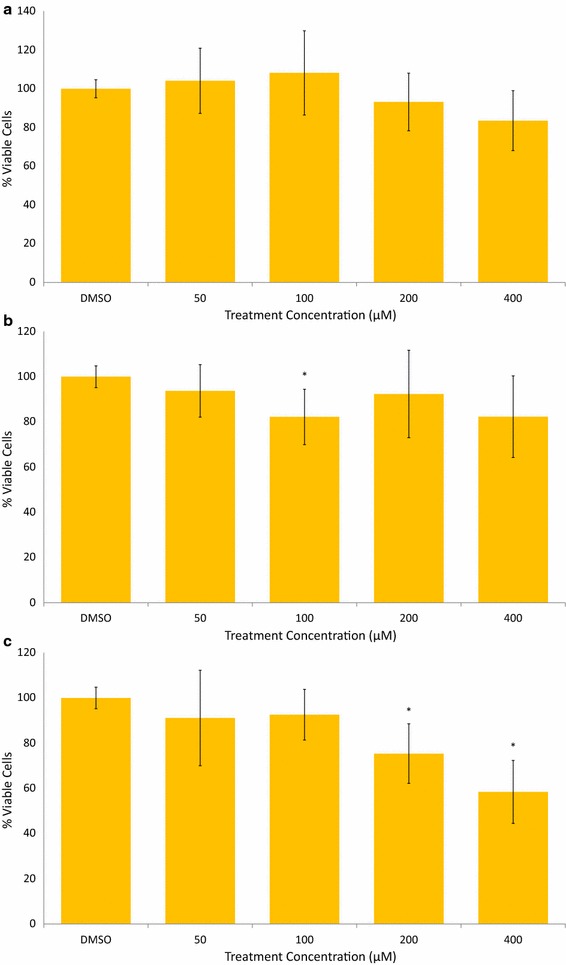
AVN-B decrease viability of MDA-MB-231 breast cancer cells in a time and concentration dependent manner. **a** AVN-B treatment, 48 h. **b** AVN-B treatment, 72 h. **c** AVN-B treatment, 96 h. Averages of a biological replicate done in triplicate; * indicates statistical significance difference from 0.4% DMSO negative control as determined by a t test at p < 0.05 level of significance, ** indicates significance level at p < 0.01, *** indicates significance at p < 0.001 level

**Fig. 4:**
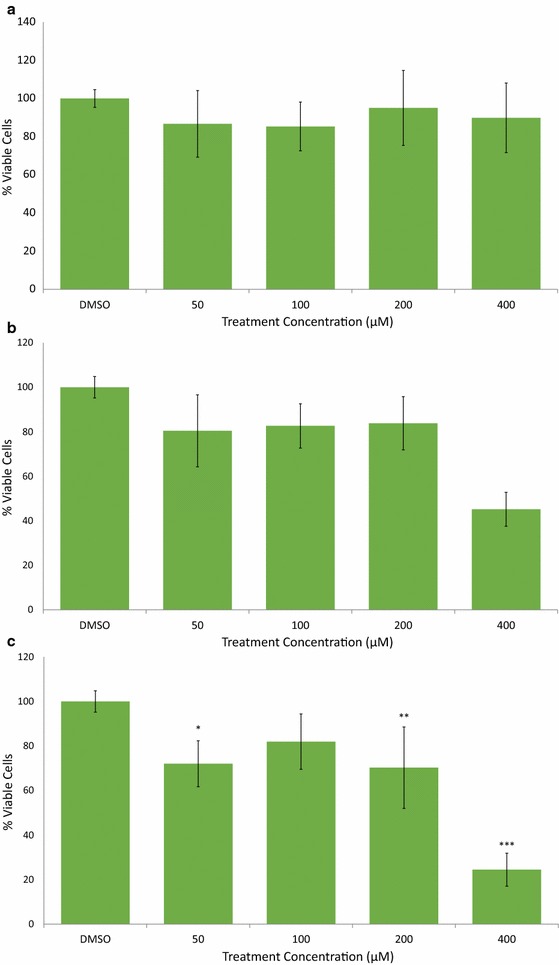
AVN-C decrease viability of MDA-MB-231 breast cancer cells in a time and concentration dependent manner. **a** AVN-C treatment, 48 h. **b** AVN-C treatment, 72 h. **c** AVN-C treatment, 96 h. Averages of a biological replicate done in triplicate; * indicates statistical significance difference from 0.4% DMSO negative control as determined by a t test at p < 0.05 level of significance, ** indicates significance level at p < 0.01, *** indicates significance at p < 0.001 level

To determine if AVN-A, B, and C have chemotherapeutic potential we first utilized an MTT cell viability assay. The effect of AVN-A, AVN-B and AVN-C on breast cancer cell viability is shown in Figs. 2, 3 and 4, respectively. Viability was assessed at, 50, 100, 200, and 400 µM treatments for AVN-A, B, and C at 48, 72, and 96 h timepoints. AVN-A was the first to show significance at the 400 µM concentration after 48 h (Fig. 2a). AVN-B and C didn’t show significance at the same concentration until the 96-h timepoint (Figs. 3c, 4c). Significant differences were noted in all three AVN treatments after 96 h with AVN-A remaining significant only at the highest dose, while AVN-B showed significance at both 200, and 400 µM and AVN-C showed significance as low as 50 µM after 96 h. Although AVN-C showed significance at a later time point than A or B, its observed decrease in viability was twice that of its counterparts. It is clear that AVN-C has a dramatic effect on breast cancer cell viability, while AVN-A and B display modest results, albeit sooner. Because of the observed differences in both timing and degree of decreased viability from the different treatments we hypothesized that different mechanisms may be at play and expected to see varying results from the treatments in further testing. Due to the fact that significance was observed in all three treatments only at 400 µM after 96 h, all following studies were conducted at this time and concentration unless noted otherwise.

### Avenanthramide-C causes DNA fragmentation

**Fig. 5:**
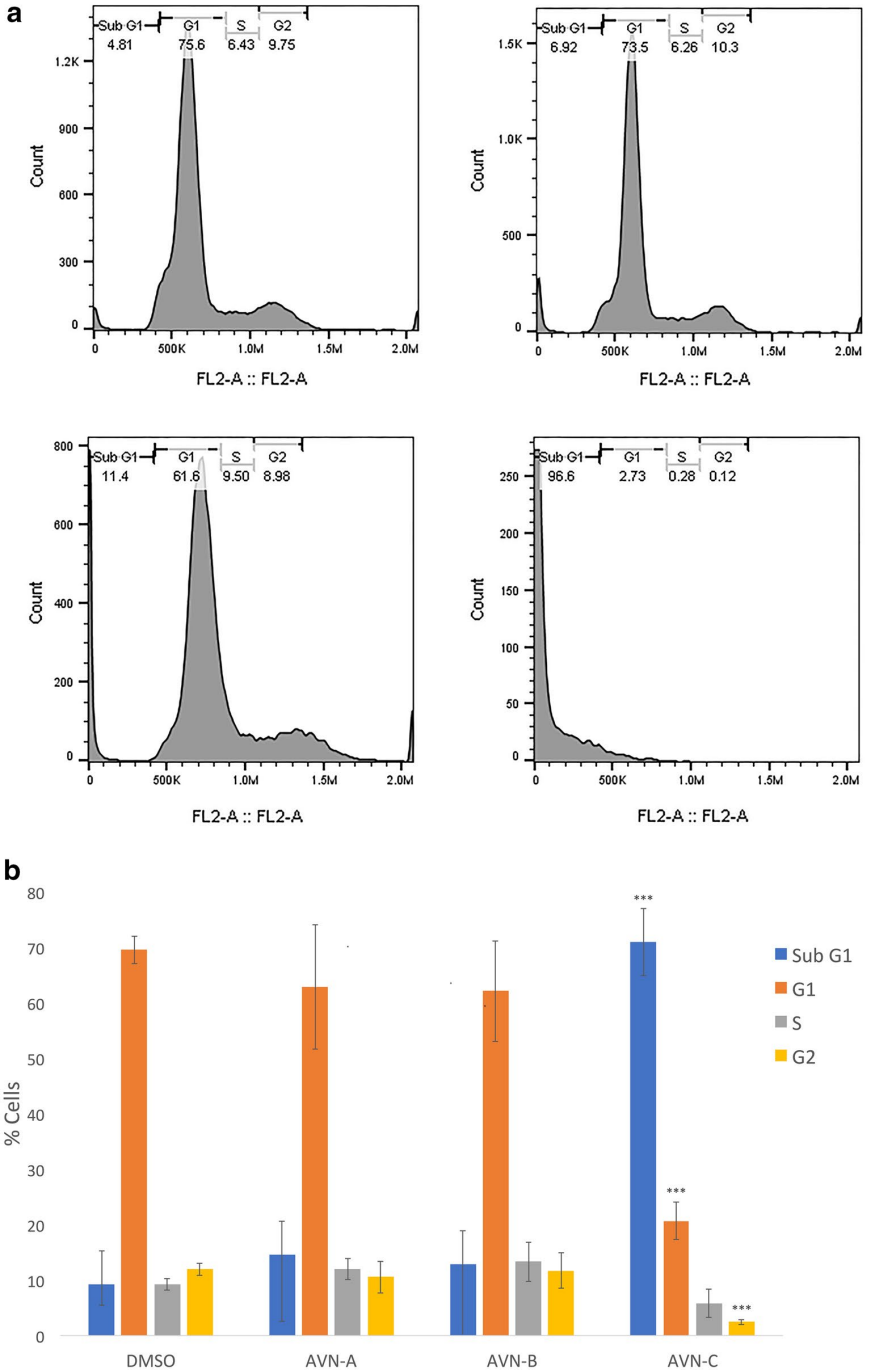
AVN-C causes DNA fragmentation and an accompanying accumulation of cells in a sub G_1_ population **a** Representative sample of flow cytometric analysis of cell cycle after fixation and staining with PI after treatment with DMSO 0.4% (vehicle) or AVN-A, B, or C at 400 µM for 96 h. **b** Graphical representation of average % cells in each stage of the cell cycle for three biological replicates done in triplicate. * Indicates statistical significance difference (when comparing each stage of the cell cycle) from 0.4% DMSO negative control as determined by a t test at p < 0.05 level of significance, ** indicates significance level at p < 0.01, *** indicates significance at a p < 0.001 level

We hypothesized that AVN may be decreasing cell viability by either inhibiting the cell cycle or activating apoptosis in the tumorigenic cells. As a result, we determined the percentage of cells in each stage of the cell cycle after 96 h of treatment with AVN at 400 µM (Fig. 5). PI staining revealed no significant difference between the vehicle and AVN-A and B for any stage of the cell cycle. Vehicle control showed 9.26% cells sub G_1_, 69.53% cells in the G_1_ stage, 9.23% cells in the S stage, and 11.97% cells in the G_2_ stage. AVN-A treatment resulted in 15.79% cells sub G_1_ (p = 0.42), 62.85% cells in the G_1_ stage (p = 0.25), 10.96% cells in the S stage (p = 0.57), and 10.4% cells in the G_2_ stage (p = 0.48). AVN-B treatment caused 14.2% cells sub G_1_ (p = 0.46), 62.49% cells in the G_1_ stage (p = 0.14), 12.75% cells in the S stage (p = 0.19), and 10.56% cells in the G_2_ stage (p = 0.60). The cells seemed to be replicating normally despite treatment and a decrease in viability. AVN-C however showed a massive accumulation of cells in a sub G_1_ population (71.03% of cells, p = 1.14E−5) resulting in significant changes to the G_1_ (20.69% of cells, p = 2.18E−6), and G_2_ (2.44% of cells, p = 2.52E−4) stages and a moderate but insignificant change to the S stage (5.85% of cells, p = 0.20).

The increase in the sub G_1_ population shows that AVN-C is causing DNA fragmentation; this is indicative of an apoptotic mechanism. It is of note that the AVN-C dependent viability decrease (75.6%) is nearly identical to the percent of cells found in the Sub G_1_ population (71%). We theorize that AVN-C works by activating apoptosis in this cell line. With no changes in cell cycle distribution for AVN-A and B their mechanism of action remained unclear. We observed an increase in sub G_1_ cells for both AVN-A and B that was accompanied by a decrease of cells in the G_1_ phase of equal magnitude, but these changes were not significant.

### Avenanthramide-C activates apoptosis in breast cancer cells

To confirm that AVN-C was activating apoptosis we conducted two experiments. First, we co-stained treated cells with PI and annexin V and second, we analyzed the caspase activity present in each sample. In both experiments we compared our treatments to a DMSO vehicle dose given at the maximum AVN treatment concentration and a treatment with a 0.75 µM staurosporine (STS), a known apoptosis activator.

**Fig. 6:**
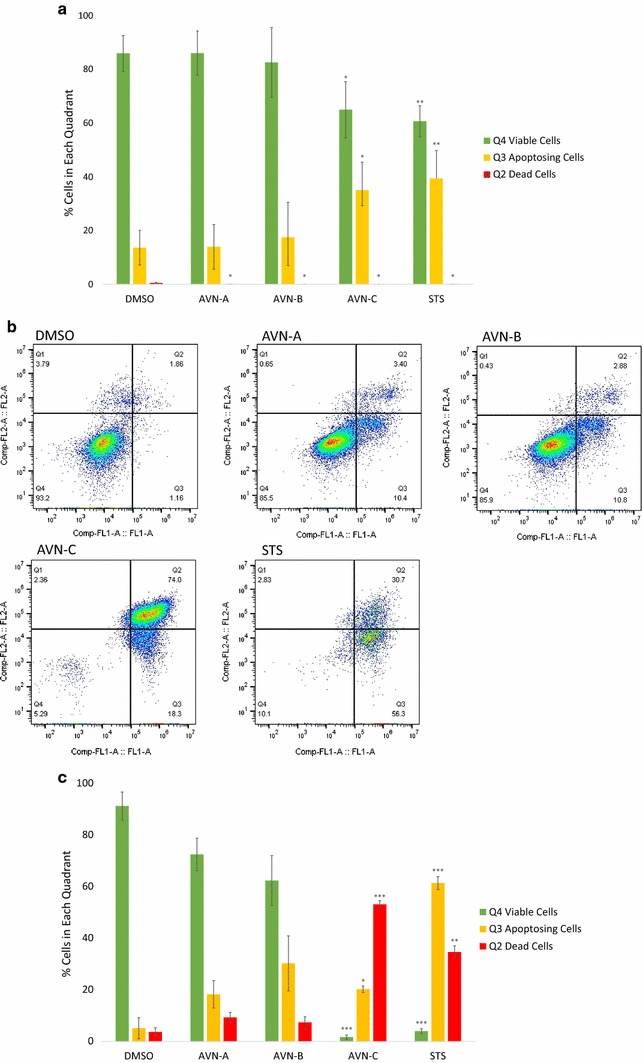
Treatment with AVN-C causes breast cancer cells to stain positive for annexin V. **a** Graphical representation of the percentage of cells in each quadrant after staining with PI and annexin V as done in biological replicate for 48-h treatment. **b** Representative sample of flow cytometric analysis of apoptosis after cells were stained with PI (Comp-Fl2-A) and annexin V (Comp-FL1-A) after treatment with vehicle DMSO 0.4%, AVN-A, B, C at 400 µM, or positive control STS for 96 h. **c** Graphical representation of the percentage of cells in each quadrant as described in (**a**). Results are a biological replicate done in triplicate for 96-h treatment. * Indicates statistical significance difference from 0.4% DMSO negative control for each individual quadrant as determined by a t test at p < 0.05 level of significance, ** indicates significance level at p < 0.01, *** indicates significance at p < 0.001 level

When staining with annexin V and PI we observed a shift from viable cells found in quadrant (Q) 4 to apoptotic and dead cells quadrants (Q3 and Q2 respectively) (Fig. 6); this occurred in all treatment groups and at both 48 and 96-h time points. After 48 h of treatment AVN-C and STS showed 35% (p = 0.04) and 39% (p = 0.0076) annexin V positive (Q3 + Q4) cells respectively, while AVN-A and B had no significant changes in annexin positive cells (Fig. 6a). At the same time, the treatment with the most cells in Q2 was AVN-A at 0.02%. However, when compared to vehicle (0.46% of cells in Q2) all trials indicated significance. When doubling the treatment time to 96 h AVN-C again had the most profound effect, leaving just over 2% of observed cells in Q4. In addition, nearly 98% of cells stained positive for the presence of annexin V (Figs. 6b, c). Of the 98% annexin positive cells, a final 27% of cells were undergoing apoptosis while 71% were dead. Taken together, these time points indicate that AVN-C induces apoptosis beginning before 48 h, and by 96 h viability has been reduced to 29 while 93% of cells still alive are on their way to an apoptotic death. At 400 µM AVN-C and 96 h we found a greater number of dead cells than after treatment with STS, indicating that AVN-C is killing cells more effectively than our positive control. All AVN-C and STS treated quadrants were significantly different than vehicle. AVN-A and B also showed marked departure from the vehicle by 96 h, in both a decrease in viable Q4 cells and an increase in apoptotic Q3 cells. Due to variance in the replicate experiments however, the p values indicated no significant difference in Q4 and Q3 as determined by a two tailed T test (0.054 and 0.08 respectively for AVN-A, and 0.092 in both quadrants for AVN-B).

**Fig. 7:**
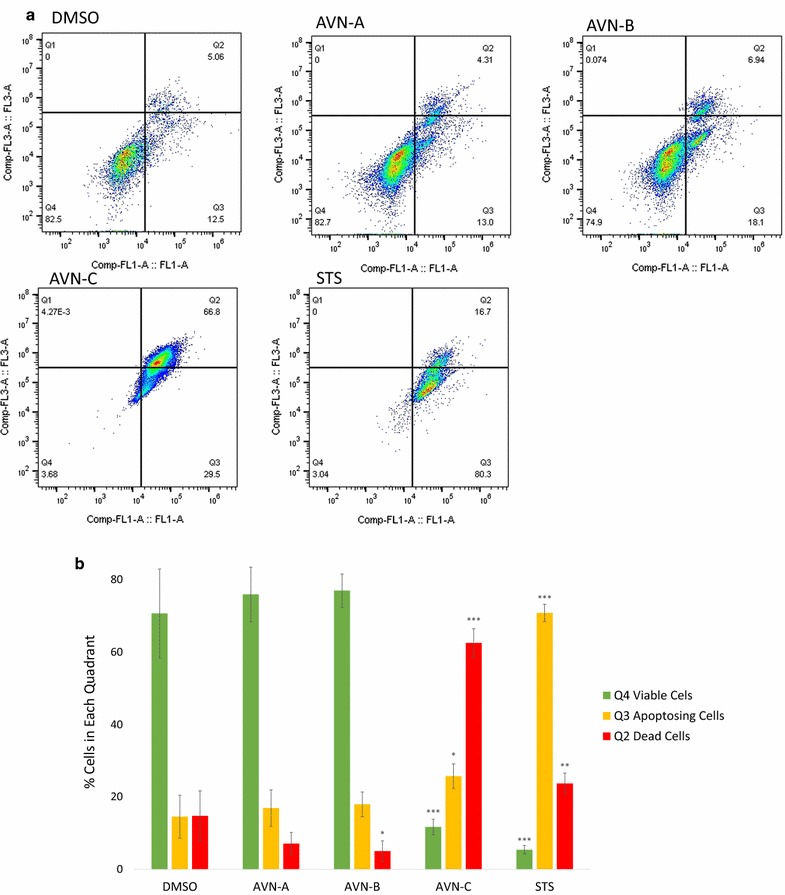
AVN-C treatment activates caspases 3/7. **a** Representative sample of flow cytometric analysis of cells treated with CellEvent Caspase-3/7 Green Flow Cytometric Assay Kit to detect caspase activity (Comp-FL1-A) and dead cells (Comp-FL3-A) after treatment with DMSO 0.4% (vehicle), AVN-A, B, C at 400 µM, or STS for 96 h. **b** Graphical representation of the percentage of cells in each quadrant after staining. Results are a biological replicate done in triplicate. * Indicates statistical significance difference from 0.4% DMSO negative control for each individual quadrant as determined by a t test at p < 0.05 level of significance, ** indicates significance level at p < 0.01, *** indicates significance at p < 0.001 level

To further confirm the apoptotic effects of AVN-C we analyzed the activity of caspases 3/7. We treated cells with each AVN and STS, but analyzed the samples to detect the activity of caspase 3/7, late stage apoptosis caspases (Fig. 7). As with the annexin V/PI co-staining, this assay allows the user to set up quadrants again detailing if cells are alive (Q4), dead (Q2), or undergoing apoptosis (Q3). For AVN-C, the results were nearly identical to those observed when staining with annexin V and PI at the same time point: 91% of cells showed caspase fluorescence and 8% of cells were found to be viable. Significance was observed in all three quadrants (p values 4.99E−8, 0.016, and 6.6E−8 for Q4, 3, and 2 respectively).

## Discussion

AVN-C decreases the viability of MDA-MB-231 breast cancer cells in vitro through an apoptotic mechanism and not due to cell cycle arrest. As a result, we suggest AVN-C should be studied further as a chemotherapeutic. AVN-A and B also show some chemotherapeutic potential, but these effects are not nearly as pronounced as those demonstrated by AVN-C, suggesting AVN-C is potentially the most effective of the three compounds in treating breast cancer.

Several plant-derived molecules have been shown to reduce cancer cell viability in a number of cancer cell lines [13]. These molecules are continually plagued by a high first pass metabolism and resulting low bioavailability, making them unfit chemotherapeutic agents [14]. AVN have high bioavailability [12] and activate apoptosis in breast cancer cells. As a result, they have the potential to act as effective chemotherapeutics. Unlike traditional chemotherapeutics AVN have not shown toxicity to normal cells; thus, AVN work as a chemotherapeutic that does not have the dose-limiting effects associated with current chemotherapy.

Chen et al. [12] demonstrated that the blood plasma levels increased by 3.3× for AVN-A, 7.3× for AVN-B, and 2.2× for AVN-C in patients when the oral dose was doubled. This data indicates that higher initial doses of AVN may significantly increase the plasma concentration for AVN. Additionally, AVN have a 3-h half-life which is much longer than many plant based compounds. The bioavailability data presented by Chen et al. indicated that the high concentration of AVN seen to be effective in this study might be obtainable in humans. Further this study has only looked at the efficacy of AVN as a chemotherapeutic, and AVN might be an effective chemopreventative compound at lower doses.

In an effort to increase the treatment effectiveness, or decrease treatment consequences, of current chemotherapy AVN-C should be investigated further. To better understand the full chemotherapeutic potential of AVN, the pro-apoptotic activity of AVN should be tested in various other cancer cell lines, specifically colon cancer (CaCo-2 and HT29) and liver cancer (HepG2) cells where decreases in viability have already been demonstrated [8, 10]. These results should also be moved to in vivo studies with various doses and delivery options for AVN-C to find IC_50_ values fit for eventual human trials.

## Conclusions

We conclude that AVN-C is a putative chemotherapeutic which causes apoptosis in MDA-MB-231 breast cancer cells. Without documented toxicity to normal cells AVN-C serves as a novel chemotherapeutic without dose-limiting side effects. It is of note that even though AVN-A and B did not activate apoptosis in a statistically significant manner, some caspase activity and positive annexin V staining was detected in our experiments. Coupled with the significant decrease observed in viability in both a time and concentration dependent manner there is much that can still be learned from these molecules and their mechanisms of action in cancerous cells.

## Abbreviations

AVN: avenanthramide
Cmax: maximum plasma concentration
PI: propidium iodide
MTT: 3-(4,5-dimethylthiazol-2-yl)-2,5-diphenyltetrazolium bromide
NP40: Nonidet P-4
DMEM: Dulbecco’s Modified Eagle Medium
FBS: fetal bovine serum
STS: staurosporine
